# Donor-dependent fecal microbiota transplantation efficacy against necrotizing enterocolitis in preterm pigs

**DOI:** 10.1038/s41522-022-00310-2

**Published:** 2022-06-09

**Authors:** Yan Hui, Gisle Vestergaard, Ling Deng, Witold Piotr Kot, Thomas Thymann, Anders Brunse, Dennis Sandris Nielsen

**Affiliations:** 1 Department of Food Science, Faculty of Science, University of Copenhagen, DK-1958 Frederiksberg C, Denmark; 2 Section for Bioinformatics, Department of Health Technology, Technical University of Denmark, DK-2800 Lyngby, Denmark; 3 Department of Plant and Environmental Sciences, Faculty of Science, University of Copenhagen, Rolighedsvej 26, DK-1958 Frederiksberg C, Denmark; 4 Department of Veterinary and Animal Sciences, Faculty of Health and Medical Sciences, University of Copenhagen, DK-1870 Frederiksberg C, Denmark; 5 Present Address: Chr. Hansen A/S, 2970 Hoersholm, Denmark

**Keywords:** Metagenomics, Microbiome, Microbiota, Next-generation sequencing

## Abstract

The development of necrotizing enterocolitis (NEC), a life-threatening inflammatory bowel disease affecting preterm infants, is connected with gut microbiota dysbiosis. Using preterm piglets as a model for preterm infants we recently showed that fecal microbiota transplantation (FMT) from healthy suckling piglet donors to newborn preterm piglets decreased the NEC risk. However, in a follow-up study using donor stool from piglets recruited from another farm, this finding could not be replicated. This allowed us to study donor-recipient microbiota dynamics in a controlled model system with a clear difference in NEC phenotype. Preterm piglets (*n* = 38) were randomly allocated to receive control saline (CON), or rectal FMT using either the ineffective (FMT1) or the effective donor stool (FMT2). All animals were followed for four days before necropsy and gut pathological evaluation. Donor and recipient colonic gut microbiota (GM) were analyzed by 16 S rRNA gene amplicon sequencing and shotgun metagenomics. As expected, only FMT2 recipients were protected against NEC. Both FMT groups had shifted GM composition relative to CON, but FMT2 recipients had a higher lactobacilli relative abundance compared to FMT1. *Limosilactobacillus reuteri* and *Lactobacillus crispatus* strains of FMT recipients showed high phylogenetic similarity with their respective donors, indicating engraftment. Moreover, the FMT2 group had a higher lactobacilli replication rate and harbored specific glycosaminoglycan-degrading *Bacteroides*. In conclusion, subtle species-level donor differences translate to major changes in engraftment dynamics and the ability to prevent NEC. This could have implications for proper donor selection in future FMT trials for NEC prevention.

## Introduction

Necrotizing enterocolitis (NEC) is a devastating intestinal inflammation primarily affecting infants born preterm with an incidence of up to 7%^1,2^. First reported in the early 1800s^3^, NEC has been intensively investigated but the underlying etiology is only partially understood^1^. In the neonatal period, the combination of impaired host antimicrobial defense mechanisms and gut dysbiosis (presence of opportunistic pathjogens and absence of commensals) might initiate a lethal hyperinflammatory reaction in the gut mucosa^4–6^. The preterm gut microbiome (GM) is often dominated by *Enterococcaceae* and *Enterobacteriaceae*^7,8^ and large-scale metagenomic studies have shown increased *Proteobacteria*^9^ and bacterial replication rates of *Enterobacteriaceae* prior to NEC incidence^10^. Although no single microbe has consistently been reported as a direct cause, accumulated evidence strongly indicates that gut microbial dysbiosis is an important risk factor for NEC.

Indeed, numerous GM-targeted interventions have been tested to prevent NEC. Examples include the use of prophylactic peroral aminoglycosides which reduces NEC incidence but is not considered feasible due to the risk of antimicrobial resistance and other potential side effects^11^. Prophylactic probiotic supplementation appears to reduce NEC incidence with few side effects^12^ and is routinely used in preterm infants, although the optimal combination of bacterial strains, dose, and timing is unknown. Regardless, the NEC incidence rate of 5–10% for very-low-birth-weight infants as well as the 10–30% mortality rate has not improved significantly the past 20 years^2,13^.

**Fig. 1:**
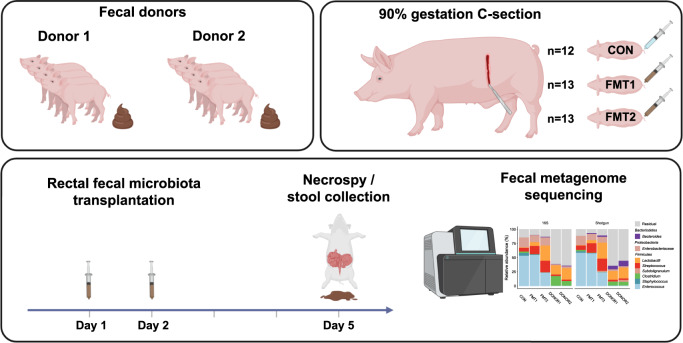
Study design. Fecal donor material was obtained from 2 different groups of term piglets (Donor 1 and 2) and rectally administered to preterm piglets born at 90% gestational age on one and two days of life. The preterm piglets were euthanized on day 5 of life. Respectively *n* = 13, 13, 12 for FMT1, FMT2 and CON. FMT1 rectal FMT with Donor 1, FMT2 rectal FMT with Donor 2, CON rectal FMT with sterile saline as control. The completed graphic was created with BioRender.com in an academic license (Supplementary Information).

Through restoring the microbial diversity in the gastrointestinal tract, fecal microbiota transplantation (FMT) has been widely and effectively used to treat recurrent *Clostridoides difficile* infection (rCDI), showing high cure rates independent of specific donors^14,15^. However, FMT therapies targeting inflammatory bowel disease (IBD) have shown discrepant clinical efficacy associated with the fecal donors^16–19^. Formula-fed preterm piglets display most clinical and pathological features of human NEC from feeding intolerance and bloated abdomen to intestinal necrosis and peritonitis^20^. This makes preterm piglets an appropriate model for microbial interventions in preterm neonates. Previously we have shown that rectal FMT can significantly reduce NEC incidence in preterm piglets while modulating the GM and host immune response with reduced mucosal proinflammatory *TLR4* signaling^21^. Accordingly, using comparable donor material we failed to reproduce the NEC-reducing effect of FMT in a follow-up experiment. This led us to hypothesize that NEC prevention by FMT depends on the donor microbiome characteristics. In this controlled experiment, we randomly allocated preterm pigs to receive FMT from either of the two fecal donors or sterile saline (as illustrated in Fig. 1). To compare gut microbial responses and investigate engraftment patterns, we characterized the gut microbiota of donors and recipients using 16S rRNA gene amplicon sequencing and shotgun metagenomics.

## Results

### Donor-dependent host response existed in recipient piglets

**Fig. 2:**
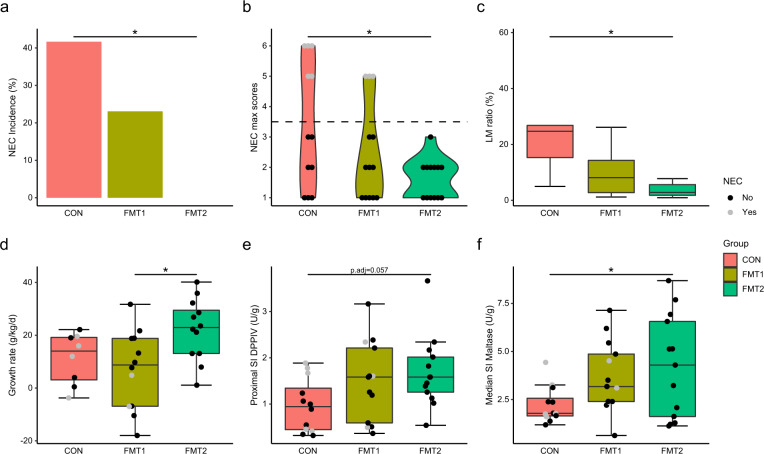
Donor-dependent host response existed in recipient piglets. FMT2 piglets showed superior survival and development status with lower NEC incidence (**a**) and necrosis severity (**b**) and improved gut barrier (**c**), daily weight gain (**d**), and digestive enzyme excretion (**e**, **f**). The label of * represents adjusted *p* < 0.05. Except for NEC incidence (two-sided Fisher’s exact test), the statistical difference was determined by Tukey’s post hoc test. Except for LM ratio and growth rate, *n* = 13, 13, 12, respectively, for FMT1, FMT2, and CON. For LM ratios, respectively *n* = 8, 8, 5 while *n* = 12, 12, 8 for growth rate. In boxplots, the inner fences are depicted as error bars. FMT1 rectal FMT with Donor 1, FMT2 rectal FMT with Donor 2, CON rectal FMT with sterile saline as control, NEC necrotizing enterocolitis, LM ratio urinary lactulose-mannitol ratio, SI DPPIV dipeptidyl-peptidase IV activity in the small intestine, SI Maltase maltase activity in the small intestine.

FMT from Donor 2 significantly reduced the NEC incidence (CON vs. FMT2, 42 vs 0%, *p* = 0.0149, Fisher’s exact test), whereas FMT from Donor 1 only resulted in a non-significant NEC reduction (Fig. 2a). The pathological severity of FMT2 piglets decreased across all gut segments (Supplementary Fig. 1a), resulting in a significant reduction in pathological severity in FMT2 piglets relative to CON (Fig. 2b). Moreover, only FMT2 had reduced intestinal permeability as indicated by a lower urinary lactulose-mannitol ratio relative to CON piglets (Fig. 2c). Furthermore, FMT2 piglets showed reduced diarrhea severity for the duration of the study (Supplementary Fig. 1b) and had higher daily weight gain relative to FMT1 (Fig. 2d), while the digestive capacity of proteins (Fig. 2e) and carbohydrates (Fig. 2f) tended to be higher relative to CON. Gut histological evaluation showed no distinct change between treatment groups (Supplementary Fig. 1c–f). However, linear regression analysis suggested that histological data was strongly related to NEC severity, where myeloperoxidase (MPO) score in the colon and bacterial adhesion score explained most variance with *R*^2^ values larger than 0.5 (Supplementary Fig. 2).

### Fecal microbiota transplantation shifted the recipient gut microbiota in a donor-dependent manner

**Fig. 3:**
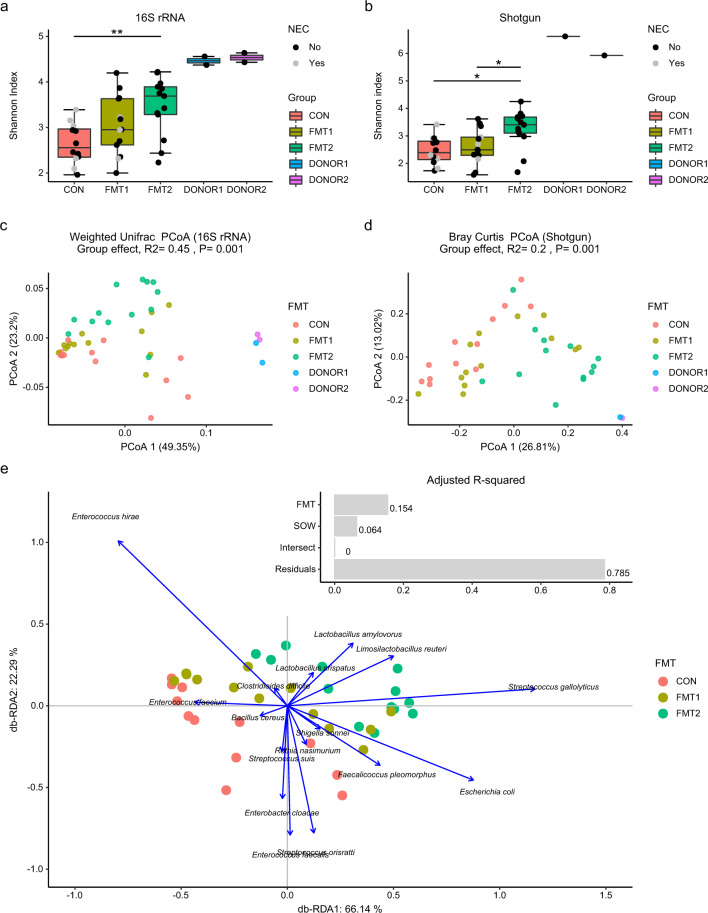
Fecal microbiota transplantation shifted the recipient gut microbiota in a donor-dependent manner. Shannon index calculated from 16S rRNA gene amplicon sequencing (**a**) and Shotgun metagenomics (**b**); Unsupervised PCoA plots based on weighted UniFrac (**c**) and Bray Curtis dissimilarity metrics (**d**), and the db-RDA biplot showing the variance explained by FMT and SOW and the scaled loadings of core species (mean relative abundance > 1%) (**e**). The labels of * and ** represent adjusted *p* < 0.05 and 0.01 (Wilcoxon rank-sum test for Shannon index and PERMANOVA for beta diversity). Respectively *n* = 13, 13, 12 for FMT1, FMT2 and CON. In boxplots, the inner fences are depicted as error bars. FMT1 rectal FMT with Donor 1, FMT2 rectal FMT with Donor 2, CON rectal FMT with sterile saline as control, NEC necrotizing enterocolitis, 16S rRNA 16S ribosome RNA gene amplicon sequencing, Shotgun Shotgun metagenomics.

To determine GM changes induced by rectal FMT, we applied prokaryotic-specific 16S rRNA gene amplicon sequencing as well as broader profiling with shotgun metagenomics. The early-life GM of preterm pigs contained more *Proteobacteria* but fewer *Bacteroidetes* when compared to the composition of both donors (Supplementary Fig. 3a). The genus-level microbial composition of the two donors was quite similar (Supplementary Fig. 3b, c), despite resulting in two distinct phenotypes in the recipients. All recipients including FMT2 piglets had lower microbial Shannon diversity in the colon when compared to either of the two donors. However, the FMT2 group showed significantly higher gut microbial richness than FMT1 and CON (Fig. 3a, b). Both sequencing methods indicated that the GM of FMT2 piglets significantly differed from FMT1 and CON (Fig. 3c, d, Table 1). Donor and sow (maternal) effects were the two main independent factors affecting GM structure, which explained 15.4% and 6.4% of the total variance in a db-RDA model (Fig. 3e). NEC status explained only 5.8% and 4.1% of GM variation on unweighted UniFrac and binary Jaccard metrics, profiled by amplicon sequencing and shotgun metagenomics, respectively (Supplementary Fig. 4).

**Table 1:** Pairwise PERMANOVA between recipient groups on dissimilarity metrics.

	WUniFrac^a^	UWUniFrac^a^	Bray Curtis^b^	Jaccard^b^	Bray Curtis^c^	Jaccard^c^
FMT1 vs FMT2	*R*^2^ = 0.093,	*R*^2^ = 0.099,	*R*^2^ = 0.13,	*R*^2^ = 0.078,	*R*^2^ = 0.15,	*R*^2^ = 0.075,
	p.adj = 0.092	p.adj = 0.007	p.adj = 0.0045	p.adj = 0.0045	p.adj = 0.01	p.adj = 0.01
FMT1 vs CON	*R*^2^ = 0.045,	*R*^2^ = 0.23,	*R*^2^ = 0.1,	*R*^2^ = 0.062,	*R*^2^ = 0.079,	*R*^2^ = 0.052,
	p.adj = 0.3	p.adj = 0.0015	p.adj = 0.008	p.adj = 0.026	p.adj = 0.082	p.adj = 0.068
FMT2 vs CON	*R*^2^ = 0.13,	*R*^2^ = 0.34,	*R*^2^ = 0.23,	*R*^2^ = 0.13,	*R*^2^ = 0.24,	*R*^2^ = 0.12,
	p.adj = 0.03	p.adj = 0.0015	p.adj = 0.003	p.adj = 0.003	p.adj = 0.003	p.ad j = 0.003

*WUniFrac* weighted UniFrac, *UWUniFrac* unweighted UniFrac, *Jaccard* binary Jaccard distance, *FMT1* rectal FMT with Donor 1, *FMT2* rectal FMT with Donor 2, CON rectal FMT with sterile saline as control.

^a^16S rRNA, dissimilarity metrics derived from 16S rRNA gene amplicons.

^b^Shotgun species, dissimilarity metrics derived from species-level profiling with Kaiju.

^c^Shotgun genus, dissimilarity metrics derived from genus-level profiling with Kaiju.

**Fig. 4:**
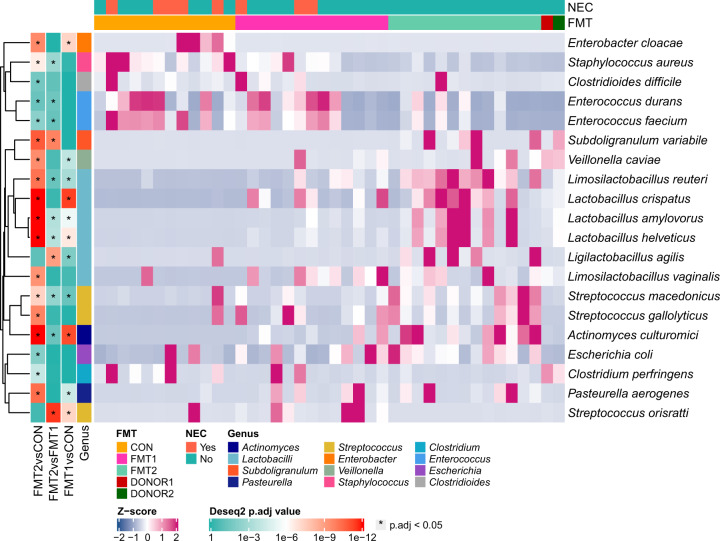
Differentially abundant taxa in colon between recipient groups. Differentially abundant core species (relative abundance > 1% among at least 10% of samples) are shown. The label of * represents adjusted *p* < 0.05 (Wald test with Benjamin-Hochberg correction). Respectively *n* = 13, 13, 12 for FMT1, FMT2 and CON. FMT1 rectal FMT with Donor 1, FMT2 rectal FMT with Donor 2, CON rectal FMT with sterile saline as control, NEC necrotizing enterocolitis. FMT, Fecal Microbiota Transplant.

*Enterobacteriaceae* and *Enterococcaceae* dominated the neonatal GM of preterm piglets. Distinct increases in lactobacilli and *Streptococcus* were observed in the colon samples of both FMT groups relative to CON (Supplementary Fig. 3b, c). Db-RDA analysis revealed that *Limosilactobacillus reuteri*, *Lactobacillus crispatus*, *Lactobacillus amylovorus* and *Streptococcus gallolyticus* contributed most of the variation induced by FMT (Fig. 3e). We adopted DESeq2 to identify species differentially abundant between treatment groups. Besides the prevalence of lactobacilli and *Streptococcus* spp., we found significant reductions in *Enterobacter cloacae*, *Staphylococcus aureus, Streptococcus orisratti*, *Clostridioides difficile* and *Enterococcus faecium* in the gut of FMT2 piglets relative to CON (Fig. 4). In the colon samples of FMT1 piglets, we observed a distinctly decreased abundance of *Lmb. reuteri*, *Lb. amylovorus*, *Ligilactobacillus agilis* and *Subdoligranulum variabile*, but the higher relative abundance of *Staph. aureus*, *E. faecium*, and *Strep. orisratti* (FMT1 vs FMT2, adjusted *p* < 0.05, Wald test with Benjamin-Hochberg correction).

### Engrafted lactobacilli strains differed according to the respective donors

**Fig. 5:**
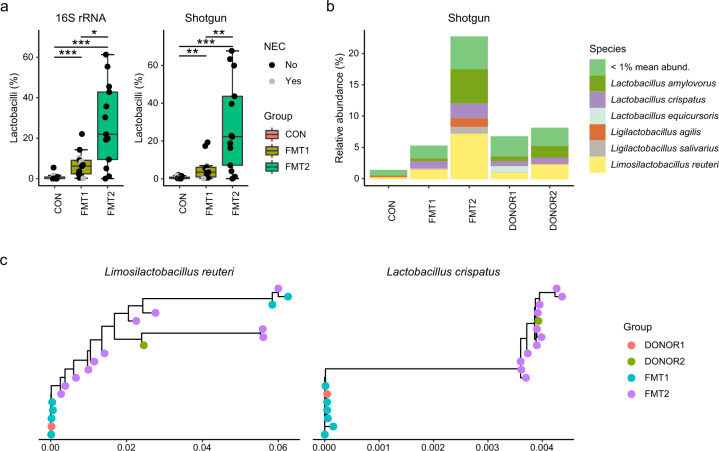
Engrafted lactobacilli strains differed according to the respective donors. The relative abundance (a) and dominant species (b) of lactobacilli, and strain tracking of *Lmb. reuteri* and *Lb. crispatus* by StrainPhlAn (c). Lactobacilli with minimum mean relative abundance of 1% are shown. The labels of *, ** and *** represent adjusted *p* < 0.05, 0.01 and 0.001 (Wilcoxon rank-sum test with Benjamin-Hochberg correction). Respectively *n* = 13, 13, 12 for FMT1, FMT2 and CON. In boxplots, the inner fences are depicted as error bars. FMT1 rectal FMT with Donor 1, FMT2 rectal FMT with Donor 2, CON rectal FMT with sterile saline as control, 16S rRNA 16S ribosome RNA amplicon sequencing, Shotgun Shotgun metagenomics.

The relative abundance of lactobacilli increased in colon samples of both FMT treatment groups, but also distinctly differed in between (Fig. 5a). *Lb. amylovorus*, *Lb. crispatus* and *Lmb. reuteri* were the predominantly detected lactobacilli species and were found in the donors as well (Fig. 5b). The *Lmb. reuteri* and *Lb. crispatus* strains in the recipients showed different donor sources when we aligned their phylogenetic marker genes (Fig. 5c). The functional difference of engrafted lactobacilli strains was compared using PanPhlAn, and 798 gene families for *Lmb. reuter*i and 432 for *Lb. crispatus* were found to be different between the strains from FMT1 and FMT2 piglets (*p* < 0.05, Fisher’s exact test). These genes were associated with ABC transporters, fatty acid biosynthesis, amino acid biosynthesis/metabolism, and drug resistance (Supplementary Data 1 and 2).

### Shifted gut microbiota composition was linked to gastrointestinal improvements

**Fig. 6:**
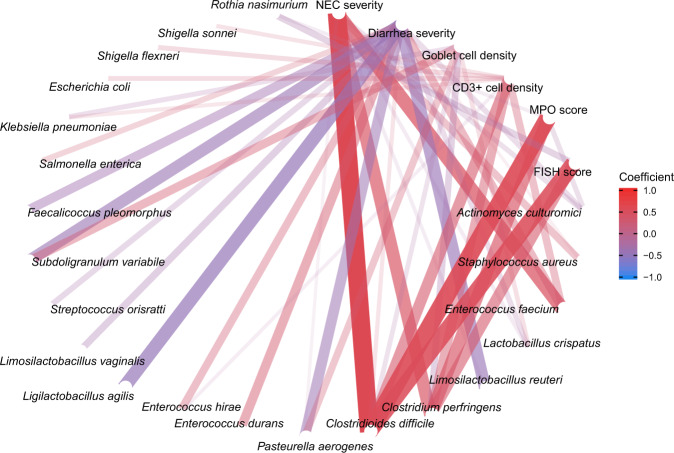
Shifted gut microbiota composition was linked to gastrointestinal improvements. The core microbiome is used for correlation analysis (relative abundance > 1% among at least 10% of samples). The average NEC and diarrhea severity are taken as the indicators for gastrointestinal health, together with histological evaluation including goblet cell density, CD3+ cell density, and MPO score in colon and FISH score in the small intestine. The correlations are shown with absolute values of coefficients higher than 0.2. NEC necrotizing enterocolitis, MPO myeloperoxidase.

In addition to the donor-dependent changes in the recipient GM, the altered microbial composition was associated with gastrointestinal improvements by FMT. Using NEC, diarrhea severity and histological data as indicators of gastrointestinal health, we conducted Pearson’s correlation analysis between the abundance of the core GM species and clinical variables. The abundances of *Cl. difficile*, *C. perfringens* and *E. faecium* positively correlated with NEC severity, MPO level and CD3+ cell density in the colon as well as bacterial adhesion score in the small intestine (Pearson’s coefficient > 0.4). Although we did not detect a direct correlation between lactobacilli and NEC severity, the diarrhea severity negatively correlated with *Lmb. reuteri*, *Lgb. agilis* and *Subdo. variabile* relative abundance (Fig. 6).

Furthermore, the shifted microbial structure led to microbial communities of differential functional capacities. We established a non-redundant pig GM gene catalog, annotated by the KEGG gene database. DESeq2 was adopted to identify differentially enriched KEGG modules between treatment groups (Supplementary Fig. 5). Compared with CON, the gut inhabitants in FMT2 piglets displayed distinct genetic enrichment involved in threonine (M00018), lysine (M00526, M00527), and acetate (M00579) biosynthesis. However, FMT1 piglets had fewer microbial genes encoding these functional capacities in the colon, but more genes associated with staphyloferrin B biosynthesis (M00875). Meanwhile, pathogenetic signatures from *Salmonella enterica* (M00856) and *Escherichia coli* (M00542) were detected in colon samples of FMT2 and FMT1 but rarely in that of CON (Supplementary Fig. 5).

### Genome-resolved analysis revealed donor-specific patterns in recipient piglets

**Fig. 7:**
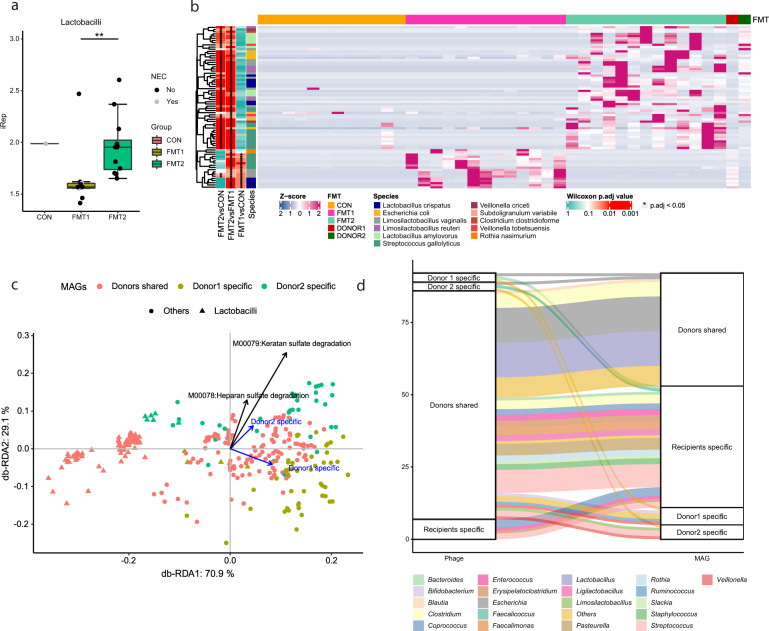
Genome-resolved analysis revealed donor-specific patterns in recipient piglets. The replication rate of lactobacilli genomes (**a**) and the abundance of engrafted MAGs (**b**) displayed donor-dependent differences. The functional similarity of donor-specific MAGs is shown by the db-RDA triplot with the scaled loadings of the glycosaminoglycans-degradation modules (**c**). Host-virus pairs are validated with spacers, where the colors of flows suggest the host taxonomy (**d**). The labels of * and ** represent adjusted *p* < 0.05 and 0.01 (Wilcoxon rank-sum test with Benjamin-Hochberg correction). Respectively *n* = 13, 13, 12 for FMT1, FMT2 and CON. In boxplots, the inner fences are depicted as error bars. FMT1 rectal FMT with Donor 1, FMT2 rectal FMT with Donor 2, CON rectal FMT with sterile saline as control.

To conduct genome-resolved analysis between donors, we refined the output of four automatic binning tools (metabat2, maxbin2, concoct, vamb) and reconstructed in total 583 medium-quality MAGs including 394 high-quality ones (completeness > 90% and contamination < 5%). Different binning strategies were compared, and the subject-specific binning strategy generated more high-quality MAGs than binning on co-assemblies (Supplementary Fig. 6). To mitigate mis-mapping bias caused by sequence homology, we used the breadth of coverage > 50% to determine the presence of a MAG in the donors. Under this criterion, piglets receiving FMT from the two donors had 199 shared MAGs, while 53 MAGs were unique to Donor 2 and 57 were unique to Donor 1. The bacterial replication rate was estimated by the sequencing coverage trend across the genome using iRep. The lactobacilli population displayed higher iRep values in the gut of FMT2 piglets relative to FMT1, while in CON iRep values were difficult to determine, due to the low relative abundance of lactobacilli (Fig. 7a). Among 309 MAGs present in donors, 74 MAGs showed differential relative abundance in the gut of FMT1 and FMT2 recipients (adjusted *p* < 0.05, Wilcoxon rank-sum test with Benjamin-Hochberg correction). Consistently with the mapping-based method, most of these MAGs belonged to lactobacilli and *Streptococcus* (Fig. 7b). The MAGs of *Lmb. reuteri, Lb. amylovorus* and *Subdo. variable* were mainly recovered in the colon samples from FMT2 piglets and Donor 2. *Lb. crispatus*, *Lmb. vaginalis* and *Strep. gallolyticus* were detected in both recipient groups but the MAGs differed according to their respective donors. Using the microbial markers provided by PhyloPhlAn, we assessed the phylogenetic relationship of the recovered MAGs. No donor-specific phylogenetic separation of the MAGs was found (Supplementary Fig. 7).

The functional similarity of donor-specific MAGs was compared based on the presence of KEGG modules in the genomes (Fig. 7c). The Donor 2-specific MAGs possessed more KEGG modules of heparan sulfate degradation (M00078, adjusted *p* < 0.005, Fisher’s exact test with Benjamin-Hochberg correction), keratan sulfate degradation (M00079, adjusted *p* < 0.01, Fisher’s exact test with Benjamin-Hochberg correction) and carbohydrate metabolism genes associated with the pentose phosphate pathway and galactose (Supplementary Data 3). Heparan and keratan sulfate are ubiquitous glycosaminoglycans (GAGs) in mammalian tissue. The two GAG-degradation modules were predominately harbored by *Bacteroides* e.g., *B. vulgatus* in Donor 2 (Breadth of coverage > 90%, Supplementary Data 4). The Donor 1-specific MAGs on the other hand showed functional enrichment in staphyloferrin B biosynthesis and drug-resistant efflux pump QacA, which likely originated from *Staphylococcus aureus* (Supplementary Data 3).

The viral genomes were categorized based on their presence in the respective donors. All vOTUs were found in the colon of recipients and approximately 95% detected in the donors, of which over 80% were shared by both donors (Supplementary Fig. 8). We compared the genetic similarity of the recovered vOTUs against the vConTACT2 reference phage genomes. A considerable number of vOTUs in the donors clustered with *Enterobacteriaceae* (e.g., *Escherichia*, *Klebsiella*, and *Salmonella*) and lactobacilli phages (Supplementary Fig. 8), corresponding to the adequate host abundance there. Using the spacer sequences, 92 host-phage matches were found between the MAGs and vOTUs, with *Escherichia*, *Streptococcus* and lactobacilli phages being most prevalent (Fig. 7d). Most host-phages identified in the pairs were shared by the two donors, except for unique phages predicted to attack *Escherichia*, *Faecalicoccus* and *Clostridiales*, which were only harbored by Donor 2.

## Discussion

FMT exhibits high cure rates (90% on average) when used to treat the overgrowth of one specific pathogen, *Cl. difficile*, in the gut^22^. The success of FMT in rCDI treatment is not affected by a specific donor^23^. In a cohort compromising of 1999 FMT recipients and 28 donors, the rCDI cure rate was found to be high for all donors^22^. Encouraged by this overwhelming success, researchers have extended the use of FMT in a range of diseases characterized by GM dysbiosis e.g., IBD^24^ and metabolic syndrome^25^. Contrarily, the super donor phenomenon appears to exist in FMT treatments of these diseases e.g., Crohn’s disease^16^, ulcerative colitis^17,18^, irritable bowel syndrome^19^ and type-2 diabetes^25^. We utilized a preterm piglet model to evaluate gut responses to FMT using two phenotypically similar donors and with a particular focus on NEC development. Using a randomized setup, preterm piglets from two litters were allocated into three treatment groups, FMT1, FMT2, and CON. Recipients of the superior donor feces (FMT2) showed no NEC cases relative to 23% NEC incidence in recipients of the inferior donor feces and 42% in the CON group. Relative to infants, NEC-like lesions of preterm piglets manifest more frequently in the ventricle and colon, and less so in the small intestine. As these gut segments are more densely populated with microbes, this could indicate that the microbial contribution to NEC development is more pronounced in piglets than infants. This model discrepancy should be considered when interpreting the data. Different animal models exist for studying NEC. Importantly, unlike the murine NEC model^26^, extra physical manipulations e.g., the cold or hypoxic stress was not introduced to induce NEC-like pathology^27^. Although the histological assessment did not show statistical differences between treatment groups, the NEC severity was strongly and positively correlated to the gastrointestinal MPO and FISH scores, as well as the CD3 + cell proportion (Supplementary Fig. 2). Besides, FMT2 piglets also exhibited improved gut function (e.g., barrier, digestion) and body weight gain relative to CON. Using a randomized design, each piglet was treated in single incubators separately to avoid possible confounding factors except the FMT donor. All in all, our data suggests the chosen donor is a determinant factor for the protective effect of FMT on preterm neonates.

A superior donor appears to be more essential for FMT use in multifactorial diseases such as IBD and NEC, which result from complex interactions between host immune response, host genetics, GM, and exogenous intake e.g., formula feeding. Gut dysbiosis is implicated in the etiology and development of these diseases but none is solely induced by overgrowth of one specific microbe as rCDI. Given the existing redox potentials in gut^28^, a preterm GM is usually characterized by relatively low microbial diversity and prolonged dominance of facultative anaerobes e.g., *Enterobacter*, *Enterococcus,* and *Staphylococcus*, but delayed colonization by obligate anaerobes e.g., *Bifidobacterium* and *Bacteroides*^29^. This might provide gastrointestinal conditions associated with high NEC risk. We noticed that the donor-dependent phenomenon was not only reflected in the host response but also in the shifted recipient GM. In the present study, the two different donors were the most important factor contributing to GM differences in the recipients, with an effect size 2–3 times higher than the second most important factor, namely the litter (Supplementary Fig. 4). Higher Shannon index and more lactobacilli but less *Cl. difficile*, *C. perfringens* and *E. faecium* were found in the colon of FMT2 piglets.

Pathogen resistance is a noted advantage provided by GM^30^, which is also expected to provide protection against NEC^31^. Using Pearson’s correlation analysis, we found the abundance of *Cl. difficile* and *C. perfringens* and *E. faecium* positively linked with increased pathological severity and gut inflammation. Although we cannot causally link these species with NEC, we still note that efficient FMT inhibited the colonization of opportunistic pathogens. Consistently, functional profiling of GM indicated the FMT2 group contained fewer microbial genes encoding staphyloferrin synthesis, but more genes associated with lysine and acetate biosynthesis in the gut. Staphyloferrin is a high-affinity siderophore excreted by *S. aureus* to acquire iron to proliferate^32^. This mechanism is employed by some pathogens to scavenge sequestrated iron from the host and plays an essential role in bacterial virulence^33^. The reduction of these genetic elements suggested a declined risk of invasive gastroenteritis by *Staph. aureus*. Lysine, considered to be an essential amino acid, has recently been found to be produced partially by GM and then absorbed by host^34^. GM-mediated amino acid metabolism could be essential for neonates given the high accretion of body protein. A disrupted GM, e.g., suppressed by antibiotics, is linked to decreased protein synthesis rate in the gut^35^. Orally administrated lysine is also reported to ameliorate diarrhea symptoms in a rodent model^36^. Besides, the superior Donor 2 resulted in recipient GM with a higher capacity for acetate production, which is in accordance with the reported increase of short-chain fatty acid production using the same donor^21^.

Donor species richness is reported to determine FMT success among IBD patients^16^. In this study, the two donor microbiotas had similar alpha diversity and composition but resulted in differential gut microbiome composition and clinical response among recipients. Hence, microbial richness alone may not ensure FMT efficiency. Our data suggest that even strain-level variation may play an important role and affect donor bacterial engraftment. The two donors had comparable lactobacilli composition; however, lactobacilli colonized recipients in a donor-dependent manner. Phylogenetic analysis indicated different strains of *Lmb. reuteri* and *Lb. crispatus* were present in the donors. Identified by PanPhlAn, *Lmb. reuteri* strains from the superior donor (Donor 2) conferred more drug resistance genes such as colistin resistance protein EmrB. The strain-level variation likely made the *Lmb. reuteri* from Donor 2 more robust to cope with environmental challenges and led to a higher replication rate of lactobacilli in the gut of FMT2 piglets. Besides, MAG-level analysis indicated that Donor 2 contained specific *Bacteroides* e.g., *B. vulgatus* conferring heparan and keratan sulfate utilization genes. Heparan and keratan sulfate are two major classes of GAGs on mammalian epithelial cells, which play a fundamental role in mutualistic bacteria adhesion^37,38^ as well as bacterial infectivity of pathogens. Given the high genome coverage of these MAGs solely in the superior donor, we suspected the GAG-degrading *Bacteroides* might competitively exclude other GAG-binding pathogens^39^. *Bacteroides* are considered as next-generation probiotics for their ubiquitous GAG-degrading capacity^37^ and production of anti-inflammatory lipopolysaccharide^40^. Gavage of *B. vulgatus* is reported to alleviate murine endotoxemia by suppressing lipopolysaccharide production in the gut^41^. One multi-donor FMT trial has also indicated that increased *Bacteroides* in donor stool is associated with ulcerative colitis remission in recipients^42^.

Although rectal FMT showed promising properties in terms of preventing NEC, we also noticed increased pathogenetic signatures from *Escherichia coli* and *Salmonella enterica* in the guts of recipients. This highlights the risk of potential transmission of infectious agents during FMT, which also explains previously high sepsis cases through oral administration^21^. Even though the transferred enteropathogens did not violate the preventive effect of rectal FMT here, they might become a risk factor^43^. Interestingly, metagenomic data confirmed the existence of adequate *Enterobacteriaceae* phages in the donors, which may act in balancing the microbiome composition through host-phage interactions. In a follow-up study, we showed that fecal filtrate transplantation could also protect preterm pigs from NEC, reduce opportunistic pathogen (e.g., *Klebsiella* spp.) colonization, and restore the balance of gut bacteria and phages^44^.

## Methods

### Animals and experimental setup

Experimental animal procedures were approved by the Danish Animal Experiments Inspectorate (2014-15-0201-00418). Thirty-eight crossbred piglets (Landrace × Yorkshire × Duroc) from two sows were delivered at 90% gestion (106 days) and fitted with an oro-gastric feeding tube and an arterial catheter inserted through the transected umbilical cord. Animals were housed separately in heated incubators (37–38 °C) with oxygen supply as previously outlined^27^. To account for the lack of transplacental immunoglobulin transfer, passive immunization was provided by arterial infusion of maternal plasma (16 ml/kg) and followed by parenteral nutrient supplementation (2–4 ml/kg/h, Kabiven, Vamin, Fresenius-Kabi, Bad Homburg, Germany). Increasing volumes of infant formula (3–12 ml/kg/3 h) were administered in the feeding tube from birth to day 5.

### Fecal microbiota transplantation

Two donor solutions were prepared from pooled colon content of four 10-day-old healthy pigs (Landrace × Yorkshire × Duroc) from two different specific pathogen-free (SPF) herds and born by multiparous sows (Fig. 1). Donor herd 1 was reared without antibiotics and donors were non-siblings, whereas donor herd 2 was conventionally reared, selected donors derived from the same litter, and all piglets were given a long-lasting Amoxicillin injection on the day of birth. All donor pigs were of an age-adequate size and with no history of infectious disease or signs of diarrhea. The donor pigs were euthanized, and colon content was collected, pooled, diluted 1:1 in 20% sterile glycerol, and stored at −80 °C^21^. All individual donor samples were free of Vancomycin-resistant enterococci and extended-spectrum β-Lactamase-producing coliforms when assessed by plating on Vancomycin-containing Slanetz-Bartley agar and Cefotaxime-containing MacConkey agar, respectively^45^. Before use, donor feces were thawed and diluted to a working concentration of 0.05 g/ml after filtration through a 70-µm cell strainer to remove macroscopic debris. Animals were stratified by gender and birth weight and randomly allocated to receive rectal administration of 0.5 ml working fecal solution from donor herd 1 (FMT1, *n* = 13), donor herd 2 (FMT2, *n* = 13), or sterile physiological saline (CON, *n* = 12). The FMT was administered twice on days 1 and 2 (one dose per day).

### Clinical evaluation and tissue collection

Animals were monitored by experienced personnel throughout the experiment, and weight and stool patterns were recorded daily. Predetermined humane endpoints resulting in immediate euthanasia included signs of systemic illness such as lethargy, respiratory distress, and hypoperfusion). On day 5, the remaining animals were euthanized with a lethal dose of intracardiac barbiturate under deep anesthesia. Three hours before scheduled euthanasia, animals received a 15 ml/kg oro-gastric bolus of lactulose and mannitol (5/5% w/v) to measure intestinal permeability by urinary recovery. After exposing the abdominal cavity, urine was collected by bladder puncture for lactulose and mannitol measurement^46^. Gross pathological evaluations of the stomach, small intestine, and colon were performed by blinded reviewers according to a validated six-grade NEC scoring system^21^. NEC was defined as pathology grade 4 (extensive hemorrhage) in at least one gastrointestinal segment, and both incidence and severity (maximum score across all three gut segments) were reported. Small intestine and colon tissues were immersion fixed in 4% paraformaldehyde and paraffin-embedded for histological assessment of bacterial adhesion by fluorescence in situ hybridization (FISH), goblet cell fraction determination by Alcian Blue-Periodic acid-Schiff (AB-PAS) staining, and T-cell and myeloid cell areas by CD3 and MPO immune staining, respectively^21^. The FISH signal was evaluated by a blinded investigator using an ordinal grading system (0–3) based on signal density and dissemination in the mucosa. Due to the high background signal, automated MPO image analysis was not possible. Instead, MPO stained tissue was evaluated using a composite ordinal grading system (0–7) consisting of cell signal density (0–3) and the extent of MPO-associated mucosa inflammation (0–4). Mucin (AB-PAS) and CD3 relative areas were quantified by image analysis using ImageJ (NIH, Bethesda, MD, USA). Representative histological staining pictures for piglets with and without NEC in the small intestine are shown in Supplementary Fig. 9. Relative to healthy (no NEC) segments, NEC samples showed eroded small intestines in a loose and incomplete epithelial structure, increased MPO activity, and bacterial aggregation in the epithelial lining. Frozen tissues were homogenized in 1.0% Triton X-100 and the homogenates were assayed for brush-border disaccharidase (lactase, maltase, and sucrase) and peptidase activities (aminopeptidases N, aminopeptidase A and dipeptidyl-peptidase IV) using a colorimetric method^46^. The enzyme activities were expressed per gram of wet intestine (proximal, middle, and distal), and one unit (U) of enzymatic activity represented the hydrolysis of 1 µmol substrate per minute at 37 °C. Colon content was collected and snap-frozen for 16S rRNA gene amplicon sequencing and shotgun metagenomics.

### DNA extraction and high-throughput sequencing

Total DNA was extracted from colon content using Bead-Beat Micro AX Gravity Kit (A&A, Gdynia, Poland) according to the manufacturer’s instructions. The V3 region of the 16S rRNA gene was amplified for prokaryotic community characterization by NextSeq 150 bp pair-end sequencing^47^ (Illumina, San Diego, CA, USA). Technical replicates of donors were sequenced for amplicon sequencing. For broader profiling, 150 bp pair-end metagenomic shotgun sequencing on Illumina Novaseq 6000 was conducted after PCR-free library preparation with KAPA HyperPrep Kit (KAPA Biosystems). The shotgun sequencing was carried out by a commercial sequencing service provider (Admera Health, South Plainfield, NJ, USA).

### Bioinformatic processing of 16S rRNA gene amplicon sequencing data

The raw reads of amplicon sequencing were merged and trimmed to construct zero-radius operational taxonomic units (zOTUs) after removing chimeras^48,49^. The Greengenes database (v13.8) was used as an annotation reference. Post analysis and visualization were performed using Phyloseq^50^. Raw zOTU data was rarefied at 11 000 counts to calculate Shannon diversity index and beta-diversity metrics (weighted and unweighted UniFrac distance metrics). For both amplicon and shotgun profiling, the genus-level taxonomic names of taxa previously belonging to the genus *Lactobacillus* were updated manually following the recently updated taxonomy for *Lactobacillaceae* by Zheng et al.^51^ When referring jointly to genera previously belonging to the genus *Lactobacillus* the term “lactobacilli” is used (as recommended in the announcement^51^).

### Bioinformatic processing for shotgun metagenomics

The bioinformatic workflow for shotgun metagenomics and integration with clinical data followed the strategy illustrated in Supplementary Fig. 10, and GNU-parallel was used to execute jobs in parallel^52^. Quality control of raw reads utilized Read_qc module within metaWRAP^53^. PhiX174 control and host genome (*Sus scrofa* 11.1, GCA_000003025.6) were removed using BMTagger and followed by Trim Galore for adapter and quality trimming, resulting in total 317 Gb clean data with a mean depth of 7.9 Gb per sample. The taxonomic profiling was done with Kaiju^54^ through protein-translated search against all prokaryotic and viral proteins in the NCBI_nr database plus microbial eukaryotic proteins (2019.6) under the default settings. Kaiju-based results were summarized at phylum, genus, and species level to calculate the respective diversity index and GM abundance, and the unclassified reads (Mean: 4.4%, Min: 0.8%, Max: 20.2%) were removed. The abundance matrix was normalized with Hellinger’s transformation before calculating the binary Jaccard and Bray Curtis distance metrics. For *de novo* assembly, each sample was individually assembled with metaWRAP^53^ Assembly module under –use-metaspades mode^55^, and then all the reads were co-assembled under –use-megahit mode^56^.

### Metagenome functional annotation

The individually- and co-assembled contigs were concatenated together for open reading frames (ORFs) prediction with MetaGeneMark^57^ (v3.38), followed by clustering with Linclust^58^ under the criteria of 95% identity and overlap > 90% (-min-seq-id 0.95, -c 0.9) to construct a non-redundant gene catalogue. Short ORFs with a length of less than 100 bp were removed with seqkit^59^. The remaining ORFs were annotated against the KEGG gene database (2019.05.15) using GhostKOALA^60^. Clean reads were mapped back to the non-redundant gene set using bwa^61^ and samtools^62^ to calculate gene abundance. Reads with mapping quality better than 30 were kept and each pair of reads mapped to the same gene counted once.

### Metagenome-assembled genomes reconstruction

To avoid fragmented assemblies of similar strains^63^, individually assembled long scaffolds (>2000 bp) were binned with maxbin2, concoct, and metabat2 in the metaWRAP^53^ Binning module. Putative bins from each sample were dereplicated using dRep^64^ (-comp 50 -con 5 –sa 0.99) to generate a medium-quality bin set (completeness > 50% and contamination < 5%) for each binner. Two-step bin refinement was adopted with the Bin_refinement module to combine results from the three binners and another deep-learning-based binner Vamb (v2.1)^65^. For Vamb, genomic bins of more than 200 000 base pairs were included. This subject-specific binning strategy extracted more complete bins compared with direct binning on co-assemblies (Supplementary Fig. 6).

The abundance of refined bins was determined by the Quant_bins module in metaWRAP^53^. The ORFs of refined metagenomic assembled genomes (MAGs) were called using prodigal^66^ under metagenomic mode (-p meta) and annotated by the KEGG gene database using GhostKOALA^60^. The breadth of coverage (covered percentage of genomic size) of MAGs in each sample was estimated by coverm (https://github.com/wwood/CoverM). The MAGs with a breadth of coverage > 50% were considered “detected” in one sample. Based on the breadth of coverage information, MAGs were grouped as “Donor1-specific”, “Donor2-specific”, “Donor shared” and “Recipients specific”. The linkage and taxonomy of the MAGs were assessed by MAGpy^67^ to integrate results from PhyloPhlAn, Checkm, and blast against the UniProt database.

To estimate the bacterial replication rate (as a surrogate measure of growth rate), the generated MAGs were first dereplicated using dRep^63^ under 99% clustering threshold (the ANImf algorithm) and 25% minimum coverage overlap, and iRep^68^ values were calculated based on the mapped reads against the representative MAGs. Default settings for iRep were adopted.

### Strain tracking of *Lactobacillus crispatus* and *Limosilactobacillus reuteri*

*Lb. crispatus* and *Lmb. reuteri* strains from the 2 donors were tracked using clade-specific marker genes from StrainPhlAn^69^ and functional gene alignment with PanPhlAn^70^. The reference assembly of *Lb. crispatus* and *Lmb. reuteri* on NCBI Refseq were downloaded to generate the respective gene database for PanPhlAn^70^. Genes on assemblies were called using Prokka^71^ and annotated against the KEGG gene database using GhostKOALA^60^.

### Viral genome identification and host prediction with spacers

The viral genome assembly and identification followed standard operating procedures^72^ using VirSorter2^73^ and checkV^74^, copped with DRAMv^75^ for manual check. The validated viral contigs (> 2000 bp) were clustered into viral taxonomic operational units (vOTUs) using Cd-dit^76^, which followed the MIUViG guidelines (>95% sequence identity and > 85% aligned coverage)^77^. These vOTUs were classified by vConTACT2^78^ to construct gene-sharing networks, visualized through Cytoscape^79^. The coverage of the vOTUs was calculated to determine the presence in the samples with Minimap2^80^ and the “jgi_summarize_bam_contig_depths” script in the MetaBAT2^81^.

The spacer sequences were mined from vOTUs using two CRISPRs detection tools CRT^82^ and CRISPRDetect^83^, and host-virus pairs were validated using SpacePHARER^84^ to check the spacer presence in the MAGs, where only identical hits were considered as correct matches.

### Statistics

All statistical analysis was performed using R version 4.1.2, and the results were visualized using R package ggplot2^85^ unless stated otherwise. The phenotypic data except for NEC incidence (two-sided Fisher’s exact test) was analyzed by linear mixed models with treatment group as a fixed factor and sow as a random factor (lmer function in R package lme4^86^), and Tukey’s *post hoc* test was used for pairwise comparison (lsmeans function in R package lsmeans^87^). For microbial diversity comparison, we used Wilcoxon rank-sum test for Shannon index and PERMANOVA (Adonis function in R package vegan^88^) for beta diversity. Benjamin-Hochberg FDR (false discovery rate) correction was adopted for multiple testing. Distance-based redundancy analysis (db-RDA) was conducted to identify the microbial variation contributed by the FMT and SOW effect using Bray Curtis dissimilarity distance. DESeq2 was adopted to identify differentially enriched microorganisms on the summarized species level and functional KEGG modules, and selected biomarkers were visualized in a heatmap by R package complexheatmap^89^. The correlation networks of microbial associations with clinical variables were calculated by Pearson’s correlation analysis after centered log-ratio transformation, and the results were visualized by igraph and ggraph (R package).

For the MAG-level analysis, we adopted pairwise Wilcoxon rank-sum tests to determine the statistical difference of MAG abundance and replication rate between groups, and the *p* values were adjusted by Benjamin-Hochberg FDR correction. Fisher’s exact test was applied to identify the differential presence of gene families and KEGG modules and between donor-specific strains and MAGs. Db-RDA was used to show functional dissimilarity of donor-specific MAGs (Bray Curtis distance based on KEGG module presence).

### Reporting summary

Further information on research design is available in the Nature Research Reporting Summary linked to this article.

## Supplementary information

Supplementary Information

Reporting Summary Checklist

Supplementary data sheet 1

Supplementary data sheet 2

Supplementary data sheet 3

Supplementary data sheet 4

## Data Availability

The raw sequence data of 16S rRNA gene amplicon sequencing and shotgun metagenomics were deposited in the Sequence Read Archive at NCBI under Bioproject PRJNA668104.

## Code availability

Code for data analysis can be accessed in the public Github repository (https://github.com/yanhui09/FMT-donor).
