# Supplementary material for: Donor-dependent fecal microbiota transplantation efficacy against necrotizing enterocolitis in preterm pigs
Source: NPJ Biofilms Microbiomes. 2022 Jun 9;8:48. doi: 10.1038/s41522-022-00310-2 (PMC9184500; doi:10.1038/s41522-022-00310-2)
Supplement: Supplementary file 1 — Supplementary Information [file 41522_2022_310_MOESM1_ESM.docx]

**Supplementary Information for:**

**Donor-dependent fecal microbiota transplantation efficacy against necrotizing enterocolitis in preterm pigs**

Yan Hui^1^, Gisle Vestergaard^2,3^, Ling Deng^1^, Witold Piotr Kot^4^, Thomas Thymann^5^, Anders Brunse^5^*, Dennis Sandris Nielsen^1^*

^1^Department of Food Science, Faculty of Science, University of Copenhagen, DK-1958 Frederiksberg C, Denmark

^2^Section for Bioinformatics, Department of Health Technology, Technical University of Denmark, DK-2800 Lyngby, Denmark

^3^Chr. Hansen A/S, 2970, Hoersholm, Denmark

^4^Department of Plant and Environmental Sciences, Faculty of Science, University of Copenhagen, Rolighedsvej 26, DK-1958 Frederiksberg C, Denmark

^5^Department of Veterinary and Animal Sciences, Faculty of Health and Medical Sciences, University of Copenhagen, DK-1870 Frederiksberg C, Denmark

*Joint senior and corresponding authors

Correspondence address

anderss@sund.ku.dk

dn@food.ku.dk

**Supplementary Figures**


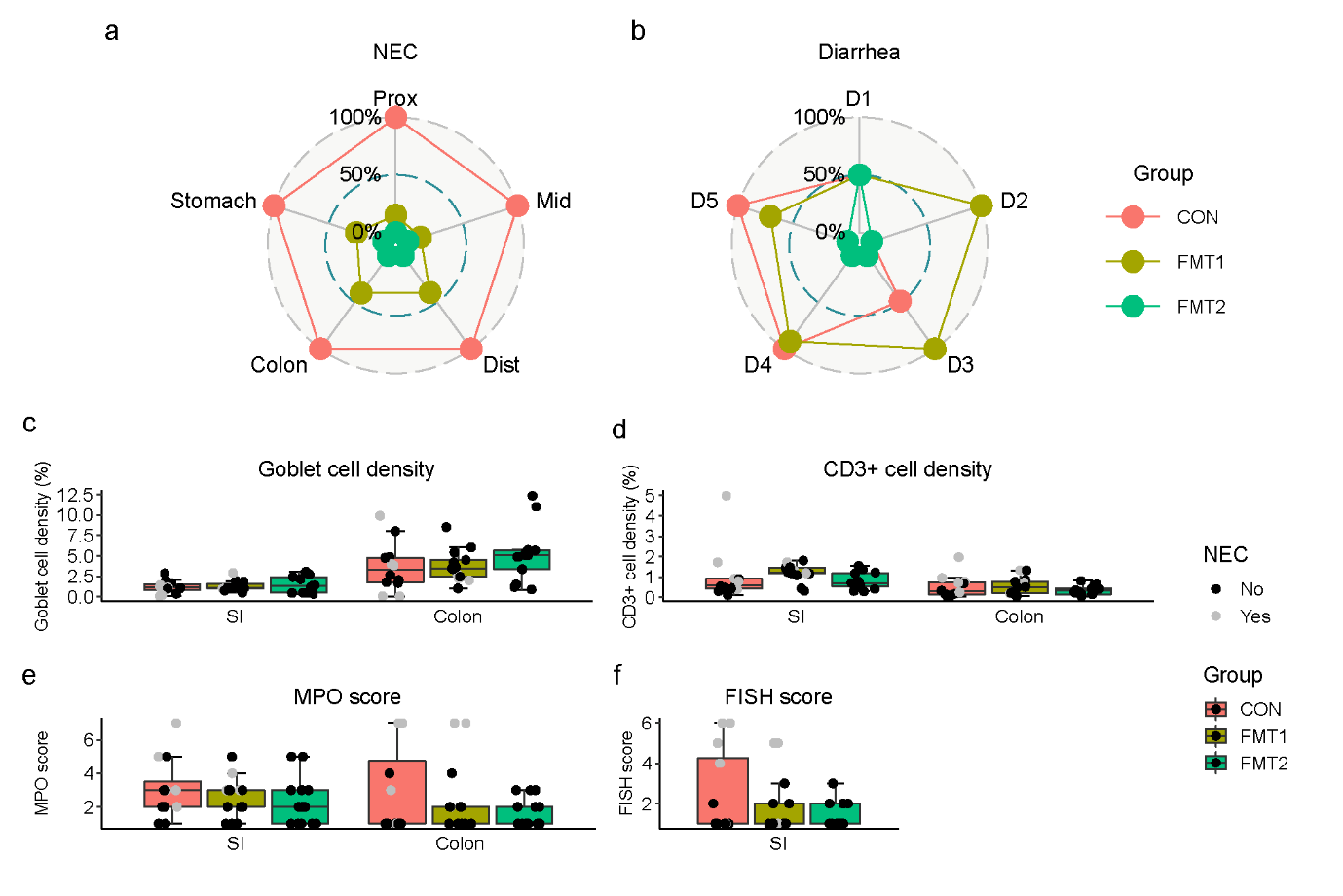
**Supplementary Fig. 1. Clinical assessment and gastrointestinal histology evaluation of recipient piglets.** Radar plots showing necrosis severity across the gastrointestinal segments (a) and diarrhea severity of recipient piglets in the first 5 days (b); Box plots showing goblet cell density (c), CD3+ cell density (d), MPO score (e), and FISH score (f) in the gastrointestinal tract. In the radar plots, data are scaled accordingly, with 0%, 50%, and 100% indicating the respective percentiles per category. In boxplots, the inner fences are depicted as error bars. Prox, Mid, and Dist represent the proximal, middle, and distal parts of the small intestine, respectively; D1, D2, D3, D4, and D5 represent 1, 2, 3, 4, and 5 days of life, respectively. MPO, myeloperoxidase; FISH, fluorescence *in situ* hybridization; NEC, necrotizing enterocolitis; FMT1, rectal FMT with Donor 1; FMT2, rectal FMT with Donor 2; CON, rectal FMT with sterile saline as control.

**
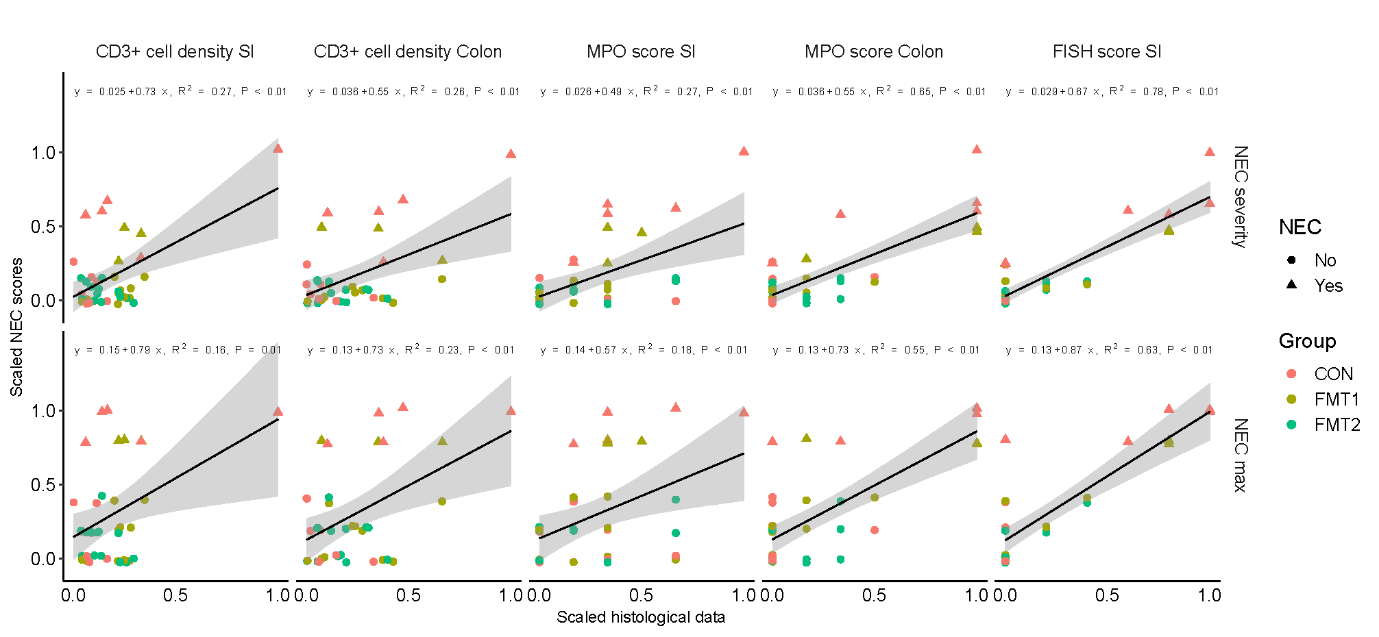
**

**Supplementary Fig. 2. Significantly positive correlations between the maximum and average NEC severity and histological data.** In the scatter plot, NEC scores and histological data are scaled to the same range between 0 and 1. NEC severity and NEC max represent the average and maximum necrosis scores across the gastrointestinal segments, respectively. SI, small intestine; MPO, myeloperoxidase; FISH, fluorescence *in situ* hybridization; NEC, necrotizing enterocolitis; FMT1, rectal FMT with Donor 1; FMT2, rectal FMT with Donor 2; CON, rectal FMT with sterile saline as control.


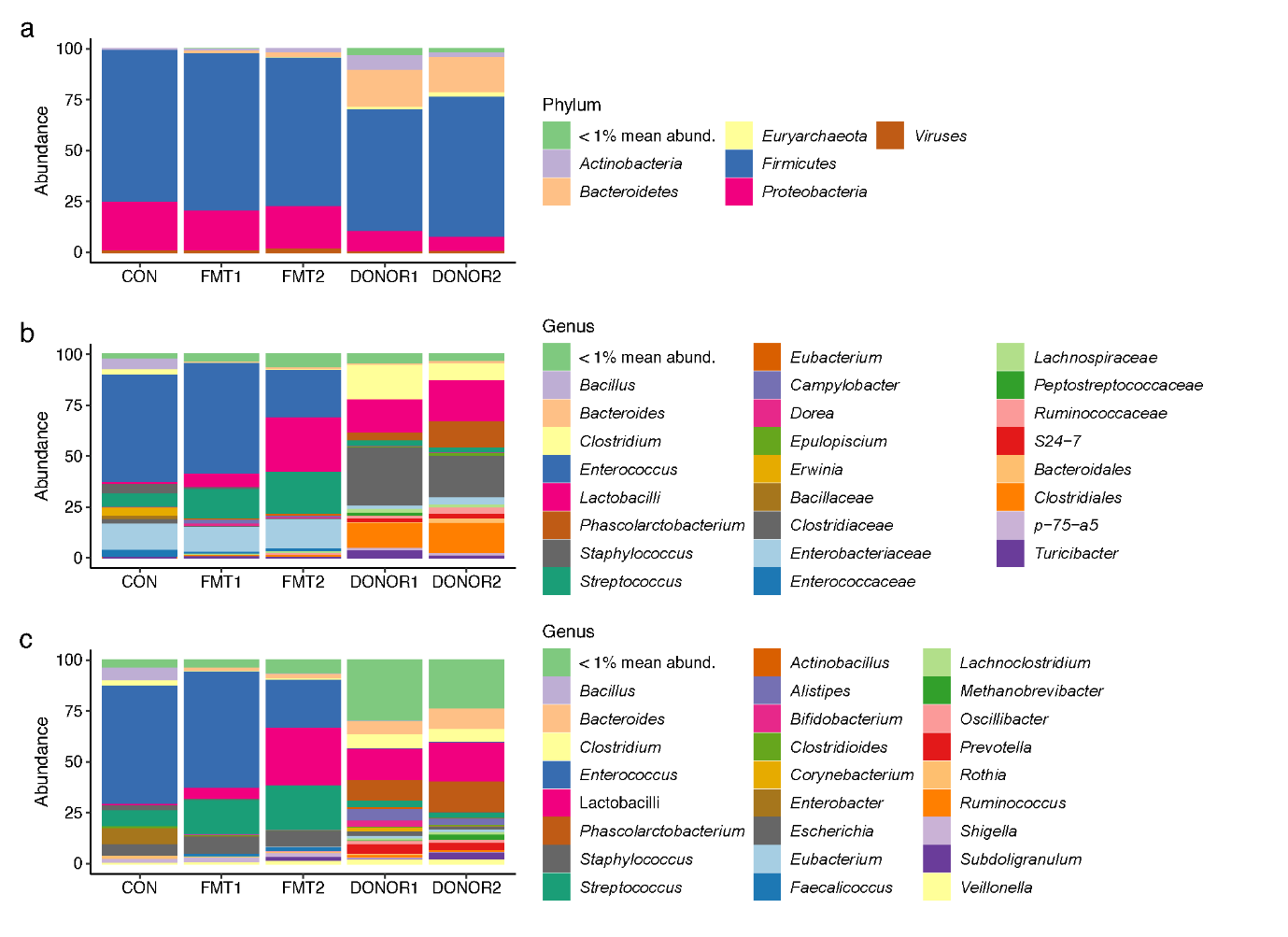
**Supplementary Fig. 3. Gut microbiome profiling of recipient piglets at phylum (a, Shotgun) and genus level (b, 16S rRNA; c, Shotgun).** Respectively *n* = 13, 13, 12 for FMT1, FMT2 and CON. FMT1, rectal FMT with Donor 1; FMT2, rectal FMT with Donor 2; CON, rectal FMT with sterile saline as control.

**
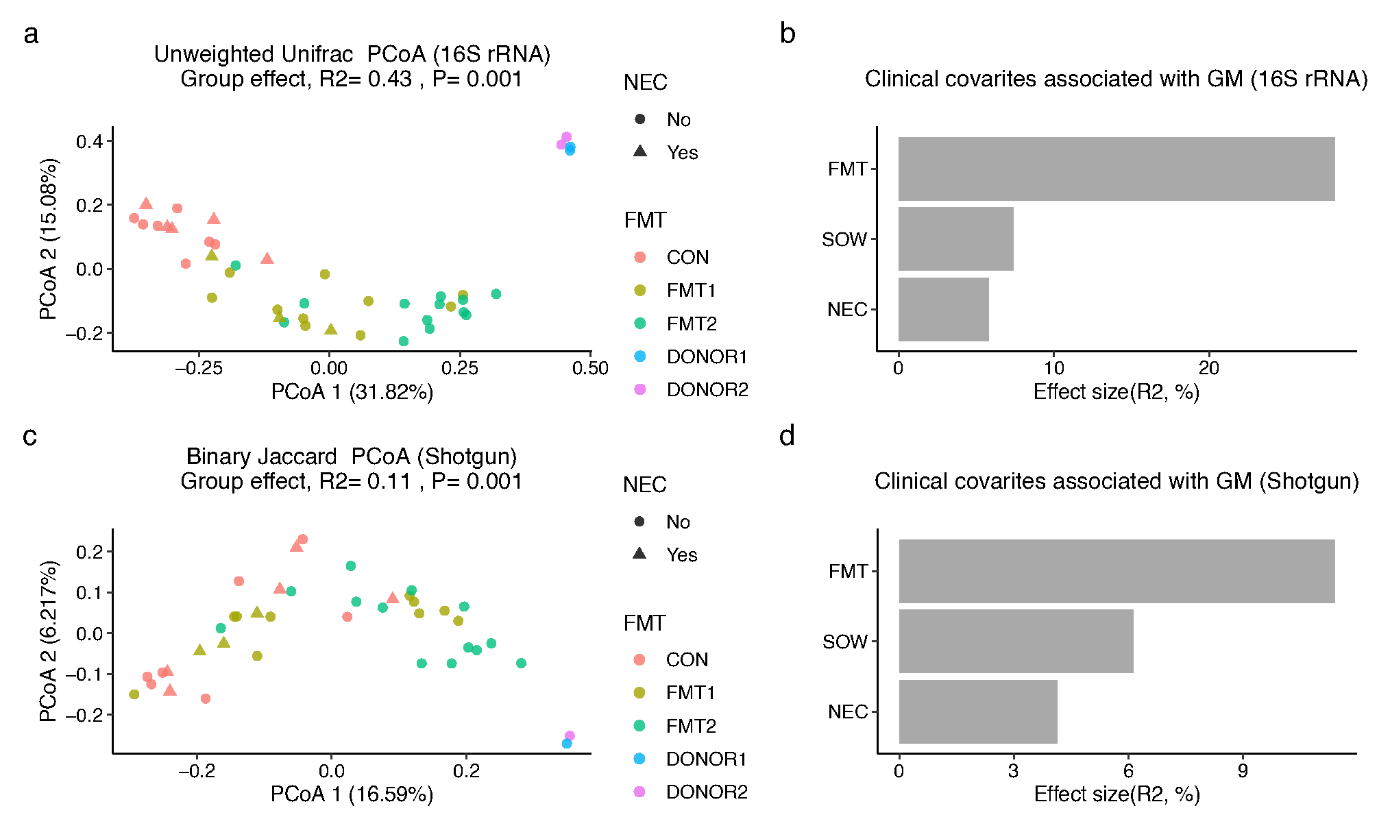
**

**Supplementary Fig. 4. Gut microbiome dissimilarity comparisons on qualitative metrics.** Unsupervised PCoA plots on unweighted UniFrac (a) and binary Jaccard distance metrics (c). The effect size of the donor, sow, and NEC was evaluated by R^2^ of PERMANOVA (b, d). Respectively *n* = 13, 13, 12 for FMT1, FMT2 and CON. FMT1, rectal FMT with Donor 1; FMT2, rectal FMT with Donor 2; CON, rectal FMT with sterile saline as control; NEC, necrotizing enterocolitis; 16S rRNA, 16S ribosome RNA gene amplicon sequencing; Shotgun, Shotgun metagenomics.


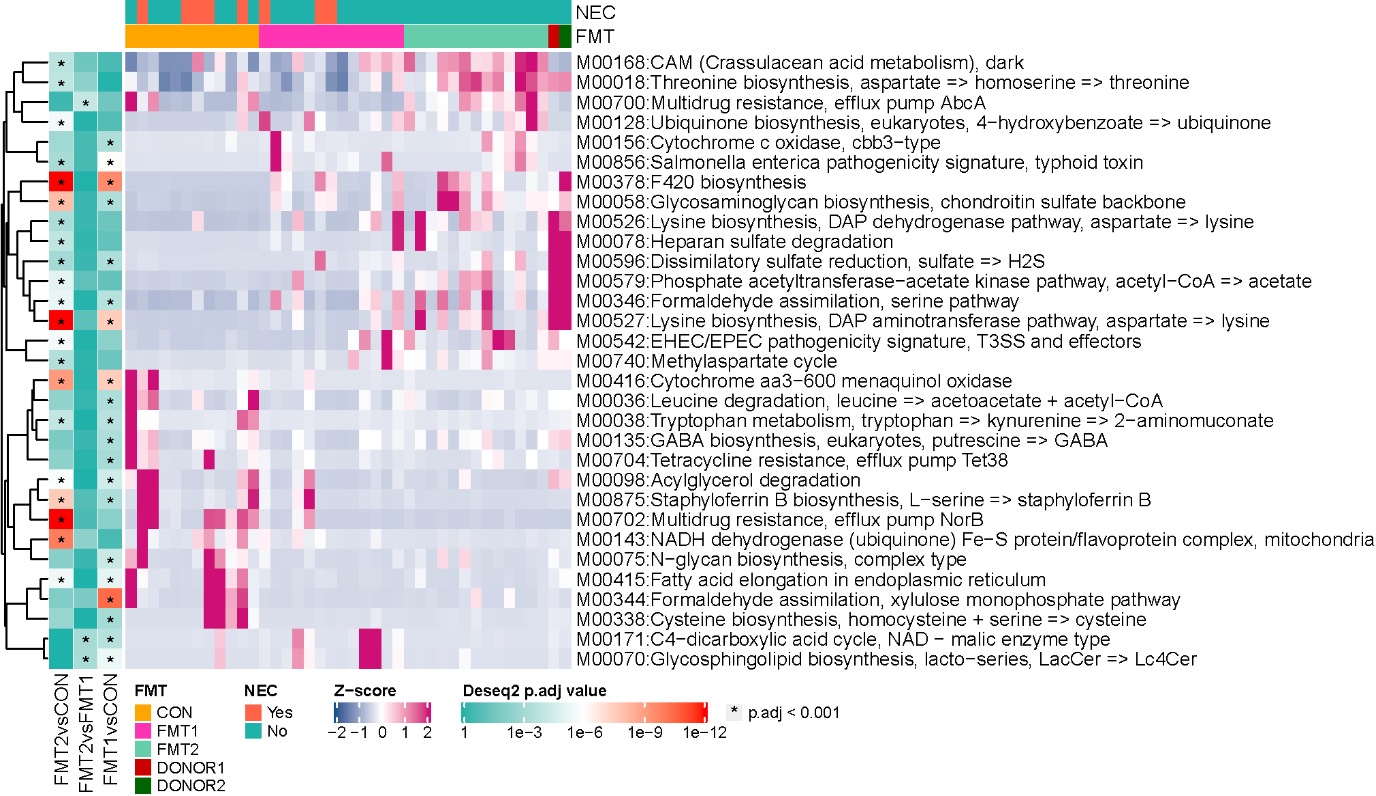


**Supplementary Fig. 5. Shifted gut microbiome functional capacity due to donor-specific FMT.** Differentially enriched functional modules are visualized. The label of * and ** indicates adjusted *p* < 0.05. Respectively *n* = 13, 13, 12 for FMT1, FMT2 and CON. FMT1, rectal FMT with Donor 1; FMT2, rectal FMT with Donor 2; CON, rectal FMT with sterile saline as control; NEC, necrotizing enterocolitis.

**Supplementary Fig. 6. Bin quality comparison of different binning strategies.** CheckM is used to assess the completeness and contamination of the reconstructed bins. CoASScoBIN, binning on co-assembled contigs; sinASSsinBIN, subject-specific binning on individually assembled contigs; Metabat2, bins from Metabat2 binner; maxbin2, bins from Maxbin2 binner; concoct, bins from Concoct binner; mmc_50_5, refined bins from Metabat2, Maxbin2 and Concoct; vamb, bins with genome size more than 200 000 bp from vamb binner; metewrap_50_5, refined bins from Metabat2, Maxbin2, Concoct and Vamb.

**Supplementary Fig. 7. The phylogeny of metagenomic assembled genomes is assessed by PhyloPhlAn.** In the phylogenetic tree, the colors of clades and rings indicate the presence of metagenomic assembled genomes in the donors, and the background colors of clades differ by their taxonomic assignment at the phylum level.

**Supplementary Fig. 8. vConTACT2 protein network clustering of vOTUs and reference virus.** The Venn diagram indicates the presence of vOTUs in the donors and colon samples of recipients. The partial network containing the discovered vOTUs is presented, and the viral genome clusters are differentiated by their respective colors and shapes. The colored dots show the viral genomes recovered in this fecal microbiota transplantation study. The top 15 phage host taxa in the reference database are shown in colored triangles.

**
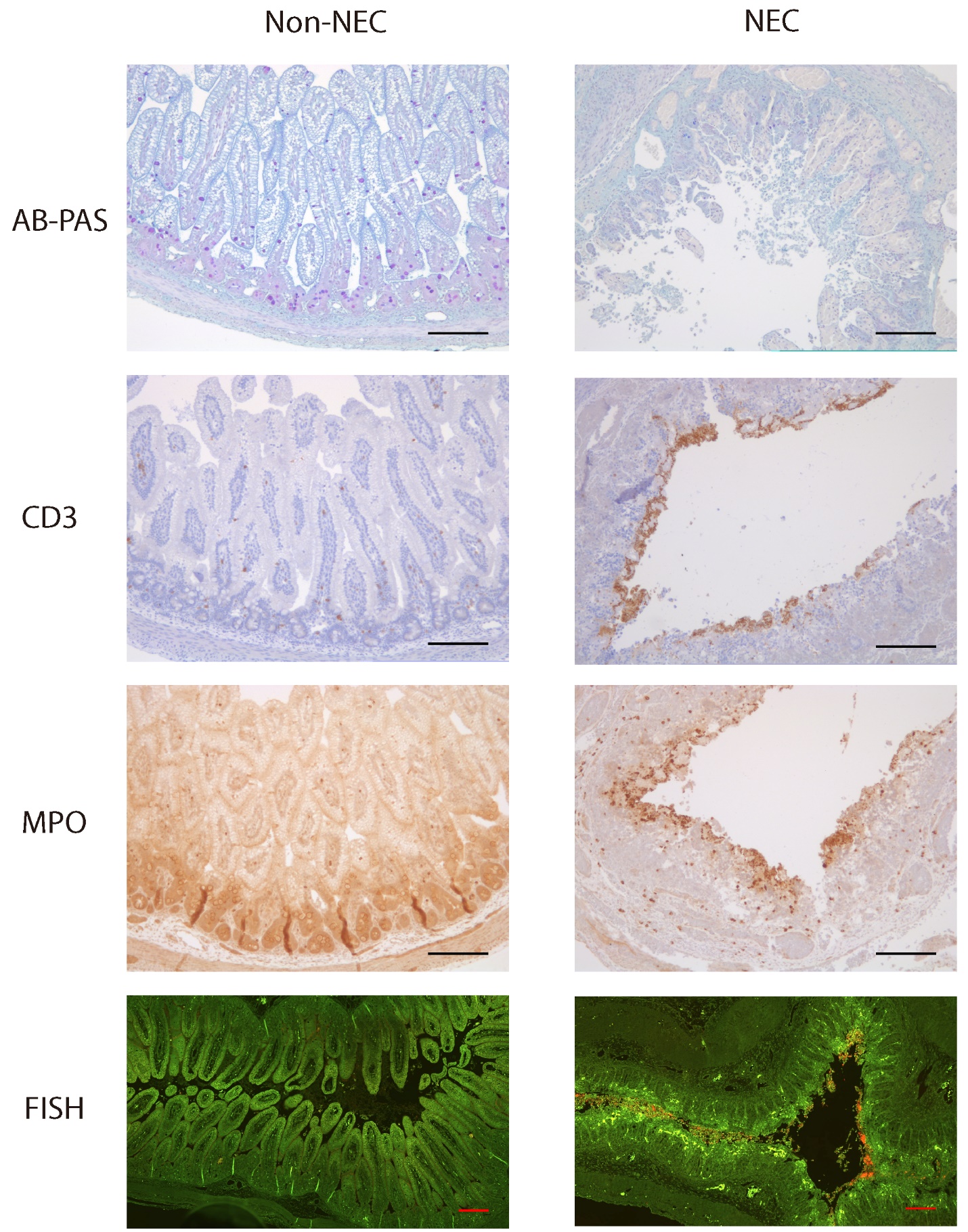
**

**Supplementary Fig. 9. Representative histological pictures of small intestine samples with or without NEC in AB-PAS, CD3, MPO, and FISH staining.** All scale bars are 200 micrometers in length.

**
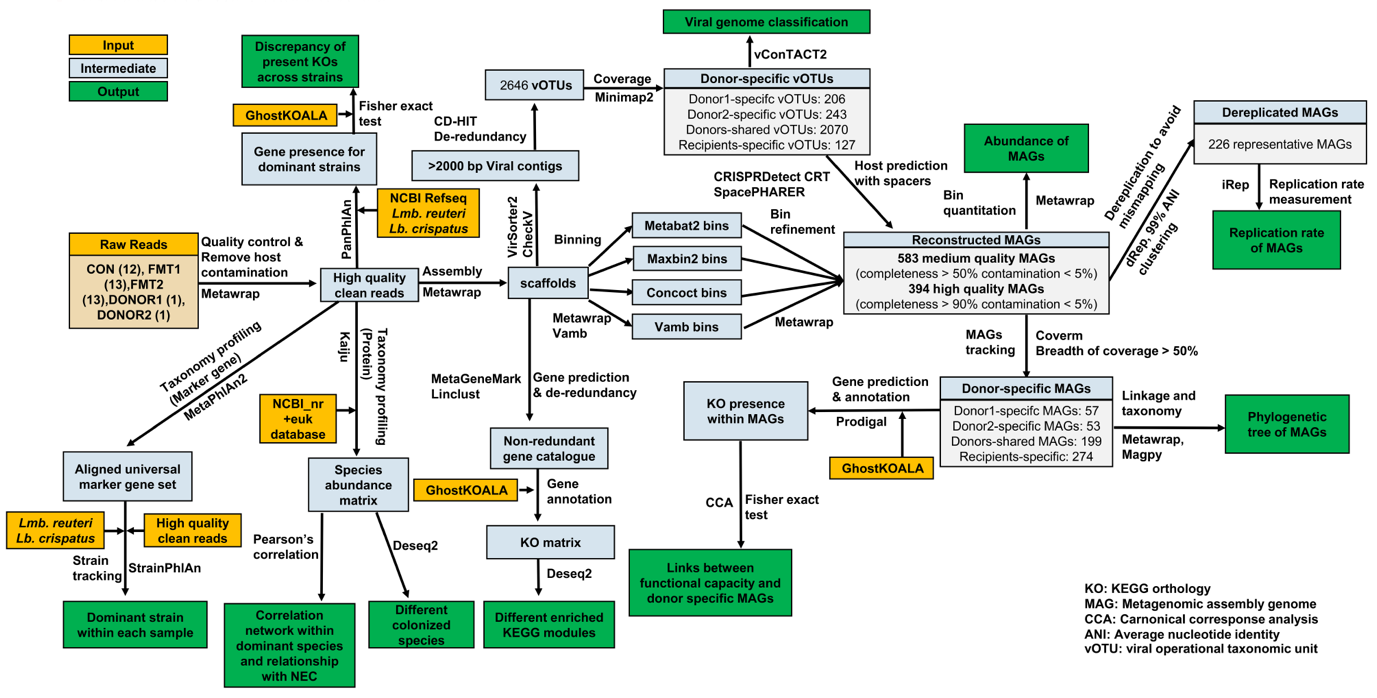
**

**Supplementary Fig. 10. Bioinformatic workflow for shotgun metagenomics.**

**Supplementary Data**

**Supplementary Data 1. Differential gene families between *Lmb. reuteri* strains identified by PanPhlAn.**

**Supplementary Data 2. Differential gene families between *Lb. crispatus* strains identified by PanPhlAn.**

**Supplementary Data 3. Differential presence of KEGG module in donor-specific metagenome-assembled genomes.**

**Supplementary Data 4. Presence of genes encoding glycosaminoglycan-degradation modules M00078 and M00079 in donor-specific metagenome-assembled genomes.**

**
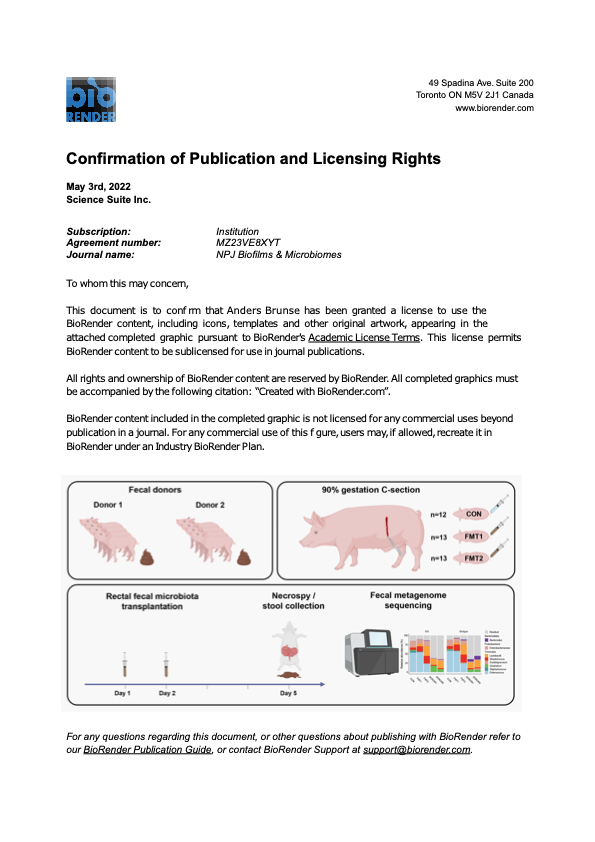
**
