# Supplementary material for: Donor-dependent fecal microbiota transplantation efficacy against necrotizing enterocolitis in preterm pigs
Source: NPJ Biofilms Microbiomes. 2022 Jun 9;8:48. doi: 10.1038/s41522-022-00310-2 (PMC9184500; doi:10.1038/s41522-022-00310-2)
Supplement: Supplementary file 2 — Reporting Summary Checklist [file 41522_2022_310_MOESM2_ESM.pdf]

nature portfolio

Corresponding author(s): Anders Brunse, Dennis Sandris Nielsen

Last updated by author(s): May 4, 2022

Reporting Summary

Nature Portfolio wishes to improve the reproducibility of the work that we publish. This form provides structure for consistency and transparency in reporting. For further information on Nature Portfolio policies, see our Editorial Policies and the Editorial Policy Checklist.

Statistics

For all statistical analyses, confirm that the following items are present in the figure legend, table legend, main text, or Methods section.

n/a	Confirmed	
☐	☒	The exact sample size (*n*) for each experimental group/condition, given as a discrete number and unit of measurement
☐	☒	A statement on whether measurements were taken from distinct samples or whether the same sample was measured repeatedly
☐	☒	The statistical test(s) used AND whether they are one- or two-sided<br>*Only common tests should be described solely by name; describe more complex techniques in the Methods section.*
☐	☒	A description of all covariates tested
☐	☒	A description of any assumptions or corrections, such as tests of normality and adjustment for multiple comparisons
☐	☒	A full description of the statistical parameters including central tendency (e.g. means) or other basic estimates (e.g. regression coefficient) AND variation (e.g. standard deviation) or associated estimates of uncertainty (e.g. confidence intervals)
☐	☒	For null hypothesis testing, the test statistic (e.g. *F*, *t*, *r*) with confidence intervals, effect sizes, degrees of freedom and *P* value noted<br>*Give P values as exact values whenever suitable.*
☒	☐	For Bayesian analysis, information on the choice of priors and Markov chain Monte Carlo settings
☒	☐	For hierarchical and complex designs, identification of the appropriate level for tests and full reporting of outcomes
☐	☒	Estimates of effect sizes (e.g. Cohen's *d*, Pearson's *r*), indicating how they were calculated

Our web collection on statistics for biologists contains articles on many of the points above.

Software and code

Policy information about availability of computer code

Data collection	No software was used in data collection.
Data analysis	Code for data analysis can be accessed in the public Github repository (https://github.com/yanhui09/FMT-donor).

For manuscripts utilizing custom algorithms or software that are central to the research but not yet described in published literature, software must be made available to editors and reviewers. We strongly encourage code deposition in a community repository (e.g. GitHub). See the Nature Portfolio guidelines for submitting code & software for further information.

Data

Policy information about availability of data

All manuscripts must include a data availability statement. This statement should provide the following information, where applicable:

- Accession codes, unique identifiers, or web links for publicly available datasets
- A description of any restrictions on data availability
- For clinical datasets or third party data, please ensure that the statement adheres to our policy

The raw sequence data of 16S rRNA gene amplicon sequencing and shotgun metagenomics were deposited in the Sequence Read Archive at NCBI under Bioproject PRJNA668104.

Field-specific reporting

Please select the one below that is the best fit for your research. If you are not sure, read the appropriate sections before making your selection.

☒ Life sciences ☐ Behavioural & social sciences ☐ Ecological, evolutionary & environmental sciences

For a reference copy of the document with all sections, see nature.com/documents/nr-reporting-summary-flat.pdf

Life sciences study design

All studies must disclose on these points even when the disclosure is negative.

Sample size	NEC incidence is a primary endpoint for this study and fecal microbiota transplantation (FMT) is assumed to reduce the necrotizing enterocolitis (NEC) incidence. The previous data (DOI: 10.1038/s41396-018-0301-z) indicated that the NEC rate was 0.125 among the preterm piglets receiving rectal FMT. The NEC incidence rate of the preterm piglet model is around 0.5 - 0.7. Assuming 0.65 as NEC rate in control group, 0.125 as that in FMT group, 0.05 as one-sided alpha, 0.8 as the commonly accepted power in animal studies, we could calculate the sample size based on Fisher's exact test. Using online tools (https://homepage.univie.ac.at/robin.ristl/samplesize.php?test=fishertest), the total sample size required is 23 on the basis of equal sample size per group. The sample size of FMT1, FMT2 and CON group is 13, 13, 12, respectively. The sample size of FMT1 or FMT2 and CON group is 25, which is above 23. Thus, the sample size is adequate to test the hypothesis.
Data exclusions	No data were excluded for analysis.
Replication	This study was replicated across two separate litters of pigs with even distribution of animals in each group. Most measures only considered biological replications (FMT1, n = 13; FMT2, n = 13; CON, n = 12) rather than technological replications. Regarding sequencing, we introduced mock community as positive control and sterile water as negative control to monitor the library preparation process. Two technological replications of the donors were sequenced in 16S rRNA gene amplicon sequencing. For each colon sample and donors, the extracted DNA were sequenced by both 16S rRNA gene amplicon sequencing and shotgun metagenomics.
Randomization	Animals were stratified by gender and birth weight and randomly allocated to receive rectal administration of 0.5 ml working fecal solution from donor herd 1 (FMT1, n = 13), donor herd 2 (FMT2, n = 13) or sterile physiological saline (CON, n = 12).
Blinding	The investigator responsible for evaluating gut pathological changes resulting in the NEC score was blinded to the intervention.

Reporting for specific materials, systems and methods

We require information from authors about some types of materials, experimental systems and methods used in many studies. Here, indicate whether each material, system or method listed is relevant to your study. If you are not sure if a list item applies to your research, read the appropriate section before selecting a response.

Materials & experimental systems

n/a	Involved in the study
☒	☐ Antibodies
☒	☐ Eukaryotic cell lines
☒	☐ Palaeontology and archaeology
☐	☒ Animals and other organisms
☒	☐ Human research participants
☒	☐ Clinical data
☒	☐ Dual use research of concern

Methods

n/a	Involved in the study
☒	☐ ChIP-seq
☒	☐ Flow cytometry
☒	☐ MRI-based neuroimaging

Animals and other organisms

Policy information about studies involving animals; ARRIVE guidelines recommended for reporting animal research

Laboratory animals	Thirty-eight crossbred piglets (Landrace × Yorkshire × Duroc) from two sows were delivered at 90% gestion (106 days) as preterm model animals.
Wild animals	The study didn't involve wild animals.
Field-collected samples	Two donor solutions were prepared from pooled colon content of four 10-day-old healthy pigs (Landrace × Yorkshire × Duroc) from two different specific pathogen free (SPF) herds and born by multiparous sows.
Ethics oversight	Experimental animal procedures were approved by the Danish Animal Experiments Inspectorate (2014-15-0201-00418).

Note that full information on the approval of the study protocol must also be provided in the manuscript.
